# An imaging approach for determining the mechanism of enhancement of intestinal absorption of an L-theanine supplement

**DOI:** 10.1371/journal.pone.0253066

**Published:** 2021-06-11

**Authors:** Yuki Sato, Kazuki Yamaguchi, Mikako Ogawa, Yoh Takekuma, Mitsuru Sugawara

**Affiliations:** 1 Faculty of Pharmaceutical Sciences, Hokkaido University, Sapporo, Hokkaido, Japan; 2 Global Station for Biosurfaces and Drug Discovery, Global Institution for Collaborative Research and Education (GI-CoRE), Hokkaido University, Sapporo, Hokkaido, Japan; 3 Department of Pharmacy, Hokkaido University Hospital, Sapporo, Hokkaido, Japan; Shenzhen Bay Laboratory, CHINA

## Abstract

**Background & objective:**

Theanine (L-glutamylethylamide) contained in green tea is a functional food component that has been attracting attention due to its relaxation effect. It was shown that the ingredients added to the theanine formulations increased the absorption of theanine. If this mechanism can be elucidated, it would be possible to contribute to development of evidence-based formulations. In this study, we investigated the effect of ingredients in the formulations on the absorption of theanine in detail.

**Main methods:**

After oral administration of a mixture of theanine and additional components to Wistar rats the plasma concentration was determined by an HPLC and the pharmacokinetic parameters were calculated. In addition, a new system for evaluating intestinal blood flow was developed since the involvement of intestinal blood flow was considered as a factor that increased absorption of theanine.

**Key findings:**

Plasma concentration of theanine increased significantly in the combined use group with eight ingredients containing piperine as compared with theanine only group. Piperine would increase theanine absorption by increased blood flow, not an inhibition of metabolism. We succeeded to develop a visual and quantitative system to evaluate the effect of these ingredients directly including piperine on the intestinal blood flow using indocyanine green while maintaining physiological conditions.

**Significance:**

Increased intestinal blood flow by these ingredients including piperine enhanced the absorption of theanine. Other mechanisms may also be considered as the mechanism by which theanine absorption is increased in addition to increased blood flow.

## Introduction

Theanine (L-glutamyl ethylamide) is present in Japanese green tea and is one of the major components of amino acids [1]. Theanine is contained not only in green tea leaves but also in other tea leaves [2]. Drinking tea containing theanine has been found to have physiological effects: a relaxing effect, generation of an α-wave in the human brain [3], and reduction in blood pressure in rats with spontaneous hypertension and in high-stress-response adults [4–7]. The neuroprotective effects of theanine quantitatively have also been focused on since theanine is a derivative of _L_-glutamic acid, which is a neurotransmitter in the brain [8,9].

As described above, theanine has been shown to have a variety of effects and it has attracted much attention among functional food components. Some studies have shown the absorption mechanism of theanine [10–12]. Theanine would be transported by the Na^+^-independent neutral amino acids system L transporter. The theanine absorption would alter due to the transporter activation or inhibition, or ingredients in the formulation. Piperine, one of the ingredients in the formulation, was then focused on. Piperine, an alkaroid that is present in the fruits and roots of *Piper longum* and *Piper nigrum linn*, is an inhibitor of glucuronidation in the liver and intestine and is widely used enhancer of biological availability to improve the bioactivity of some components [13]. Glucuronidase would not be involved in theanine metabolism. It was considered that this increase of absorption of theanine would not be derived from an inhibition of metabolism by piperine. Piperine has also been reported to modulate permeation properties of the intestine by causing alteration in membrane dynamics [14]. However, this report did not directly show the effects of piperine itself. Elucidation of the mechanisms is important for the development of formulations based on scientific evidence.

In this study, the effect of the ingredients in the formulation on the absorption of theanine was investigated. In addition, since changes in blood flow were considered about the increase of plasma concentration of theanine, a new method for detecting change in blood flow visually was developed and analyzed in order to clarify the mechanism. This is the first report showing that piperine increases blood flow in the intestinal tract visually while maintaining a physiological condition. This system could be used to apply to detect the effects of other components on blood flow.

## Materials and methods

### Chemicals

Theanine powder and 8 ingredients for theanine formulations were kindly donated by FANCL Corp. (Kanagawa, Japan). Eight kinds of ingredients are as follows: *Piper longum*, creatine, proteoglycan, α-lipoic acid, cyanocobalamin, zanthoxyli fructus, pyridoxine hydrochloride and folic acid. 2-Aminobicyclo-(2,2,1)-heptane-2-carboxylic acid (BCH) and leucine were purchased from Sigma-Aldrich Co., LLC (St. Paul, MN) and Kyowa Hakko Kogyo Co., Ltd. (Tokyo, Japan), respectively. Indocyanine green (ICG) (Diagnogreen^®^) was purchased from Daiichi-Sankyo Co., Ltd. (Tokyo, Japan). Other major reagents were purchased from Wako Pure Chemical Industries, Ltd. (Osaka, Japan). All of the reagents were of the highest grade available and used without further purification.

### Animals

Male Wistar rats, aged 5 or 6 weeks (160–180 g in weight), were obtained from Jla (Tokyo, Japan). All of the rats were housed in plastic cages. The housing conditions were the same as those described previously [15]. The experimental protocols were reviewed and approved by the Hokkaido University Animal Care Committee in accordance with the “Guide for the Care and Use of Laboratory Animals (approval number 18–0044)”.

### Cell culture

Caco-2 cells obtained from RIKEN (Ibaraki, Japan) were maintained in a plastic culture dish (AS ONE, Osaka, Japan) as described previously [16]. The cells were given a fresh medium every 2 days. When the cells had reached confluence after 4–6 days of culture, they were harvested with 0.25 mM trypsin and 0.2% EDTA in phosphate buffer saline (PBS; 137 mM NaCl, 2.68 mM KCl, 8.10 mM Na_2_PO_4_, 1.47 mM KH_2_PO_4_), resuspended, and seeded into a new dish.

### Preparations of theanine, cephalexin formulations and ICG and piperine solution

Four kinds of theanine formulations were prepared as follows: theanine powder, theanine and a mixture of 8 ingredients, theanine and *Piper longum*, and theanine and a mixture of 7 ingredients (excluding *Piper longum*). All of the formulations contained 20 mg of theanine per milliliter. On the other hand, two kinds of cephalexin formulations were also prepared as follows: cephalexin powder, cephalexin and a mixture of 8 ingredients. These formulations contained 20 mg of theanine, or 5 mg of cephalexin per milliliter, respectively. The content of the additives in 1 mL containing a mixture of 7 or 8 ingredients in the theanine or cephalexin formulation is as follows: 100 mg of creatine, 60 mg of *Piper longum*, 20 mg of proteoglycan, 2.35 mg of α-lipoic acid, 0.80 mg of cyanocobalamin, 0.70 mg of zanthoxyli fructus, 0.50 mg of pyridoxine hydrochloride and 0.10 mg folic acid.

ICG solution (0.2 mg/mL water for injection) was prepared. Sodium heparin (1,000 U/mL) was diluted with saline 100 times, and a solution of saline and heparin was obtained. The solubility of piperine itself to water is low. Piperine solution was then suspended in a mixture of ethanol/propylene glycol/distilled water = 1/7/30 (v/v/v) based on previous reports (5 mg/mL, dose: 10 mg/kg weight) [17,18].

### Oral administrations and collection of samples

To obtain data of plasma concentration profiles after oral administration of theanine or cephalexin, a total of 32 rats were used to detect statistical differences. The rats were fasted for 14–16 h before the experiments. Theanine (dose: 20 mg/kg weight) as a 2% solution (1 mL/kg) or cephalexin (dose: 10 mg/kg) as a 0.5% solution (2 mL/kg) was orally administered using a gastric sonde tube. The concentrations of theanine or cephalexin and other ingredients in each formulation excluding the formulation without piperine are all the same. Blood samples were collected and plasma samples were obtained as described previously [19]. All of the samples were kept at -20°C until the assay.

### Injection and observation of ICG

For data of time profile of intestinal blood flow, a total of 12 rats were used to be able to detect statistical differences. The rats were fasted for 14–16 h before the experiments. They were anesthetized by inhaled isoflurane [20] with 2.5–3.0% using laboratory animal anesthesia device SN-487 (Shinano manufacturing Co., Ltd., Tokyo, Japan). A small midline incision was made in the abdomen and the upper small intestine (15 cm) was radially exposed. A bile duct cannulation with a tube (0.5 μm tube, PE, Becton Dickinson, USA) for drainage of ICG was filled with heparin sodium (1,000 U/mL). ICG was administered by constant intravenous infusion from the tail vein at 5 mL/h with a syringe driver (TE-331S, TERUMO Corp, Tokyo, Japan). When administered intravenously, ICG is distributed only in blood and is excreted in bile. It was confirmed that the steady state reached 15 min after the start of injection of ICG. Fluorescence time lapse imaging was performed for 30 sec with pde-neo C10935-20 (Hamamatsu Photonics, K. K., Shizuoka, Japan) as the reference for the individual. The conditions used were as follows: excitation wavelength, 760 nm; emission wavelength, 830 nm; brightness, 4.5; contrast, 2.0; excitation light intensity, 3.0. Fluorescence intensity was set to the control level (intensity before administration of theanine and/or some ingredients). The test solution was then injected into the exposed intestine. After injection of the test solution, time-lapse imaging was performed up to 60 min. Three or more of the three-branched part of blood vessels present in the mesentery were selected for objective evaluation of fluorescence intensity in each rat. The fluorescence intensity at each part was quantified with ImageJ^®^ for 30 sec from the start of imaging. The average value of 3 or more parts was used as an index of blood flow of the individual at the time point. The ratio of fluorescence intensity was then calculated with regards to the control (before the administration of test solution). Each rat was kept on a heater at about 37°C and the intestine was covered with sterile plastic wrapping and a humidifier with saline to prevent tissue from drying during the experiment except during fluorescence imaging.

After each experiment, rats were euthanized with excess sodium pentobarbital in accordance with the “Guide for the Care and Use of Laboratory Animals”. There were no animals who were dead or became severely ill during any studies.

### Uptake study in Caco-2 cells

For uptake study, Caco-2 cells were seeded at a cell density of 1.0–2.0 × 10^5^ cells/cm^2^ on a 24-well plastic plate (Corning Costar Inc., NY). The cell monolayers were given a fresh medium every 2 days and were used at 4–6 days for the uptake experiments. After removal of the growth medium, 0.5 mL of incubation buffer was added to wash each cell monolayer twice and 0.5 mL of incubation buffer containing theanine solution with or without BCH and leucine was added. The incubation buffer was the same as that described previously [21]. The monolayers were incubated for the indicated time at 37°C. Each cell monolayer was rapidly washed twice with 0.5 mL ice-cold incubation buffer without a substrate at the end of the incubation period. Two hundred microliters of distilled water per well was added and incubated for 20 min at -80°C to break down the cell membrane. One hundred and twenty microliters of acetonitrile and 20 μL of ε-aminocaproic acid (final concentration of 27.8 ng/mL) as an internal standard were added to 100 μL of a sample, and the mixture was shaken. After centrifugation at 15,000 × g for 20 min at 4°C, the supernatant was taken as a sample for LC/MS injection. Uptake values were corrected against the protein content. The protein content was measured by the method of Lowry et al. with BSA as a standard [22].

### Analytical procedures

The plasma concentration of theanine was determined using an HPLC system equipped with an L-7100 pump and an L-7485 FL detector (HITACHI, Tokyo, Japan) based on a previous report with some modifications [23]. Five hundred microliters of distilled water and 100 μL of 200 μM ε-aminocaproic acid were added to 50 μL of a sample, and the mixture was shaken for 30 s. Then 150 μL of acetonitrile was added and the mixture was shaken. After centrifugation at 18,800 × g for 5 min at 4°C, 20 μL of the supernatant was taken and 180 μL of 0.2 M sodium borate buffer (pH 9.0) and 60 μL of 10 mM NBD-F (in acetonitrile) were added and the mixture was incubated for 40 min at room temperature for derivatization. Immediately, 240 μL of 50 mM hydrochloric acid was added to stop the reaction and samples for HPLC injection were obtained. The column for HPLC was a GL Sciences Inertsil^®^ ODS-4 (3 μm in particle size, 3.0 mm in inside diameter × 150 mm) (Tokyo, Japan). The gradient elution was applied using 75 mM H_3_PO_4_/acetonitrile = 84/16 (v/v) (A) and 50 mM KH_2_PO_4_/acetonitrile/methanol = 40/21/39 (v/v/v) (B) as the mobile phase and programmed as follows: the gradient started with 100% of eluent A for 22.5 min and was decreased linearly down to 20% for 7.5 min. Then, it was decreased linearly again down to 0% for 15 min. This composition was held for a further 7.5 min before returning to 100% of eluent A immediately followed by re-equilibration for 7.5 min. Column temperature and flow rate were 30°C and 0.48 mL/min, respectively. The wavelengths of excitation and emission for detection were 470 and 540 nm, respectively. Twenty μL of a sample was injected into the HPLC system.

Chromatographic separations of uptake of theanine in Caco-2 cells were performed using an Acquity UPLC Quattro premier XE tandem quadrupole mass spectrometer (Waters Corp., Milford, MA, USA) with a COSMOSIL^®^ HILIC packed column (3 μm in particle size, 3.0 mm in inside diameter × 150 mm) (Nakalai Tesque Inc., Kyoto, Japan) based on a previous report with some modifications [24]. The gradient elution was applied using acetonitrile (A) and 10 mM ammonium acetate (B) as the mobile phase and programmed as follows: the gradient started with 70% of eluent A for 6 min and was decreased linearly down to 30% for 9 min. Then, it was returned linearly again up to 70% for 5 min for re-equilibration for 7.5 min. Column temperature and flow rate were 30°C and 0.4 mL/min, respectively. Five μL of a sample was injected into the UPLC system.

### Statistical analysis

Some pharmacokinetic parameters of theanine were analyzed. The area under the curve (AUC) was calculated by the trapezoidal rule. T_1/2_ (half life) and K_e_ (elimination rate constant) were calculated by the following formulae.


$$Log\;C=-\;\frac{Ke}{2.303}t+\text{log}\;C_{0}\ldots$$ (1)

$$T_{1/2}=\frac{0.693}{Ke}\ldots$$ (2)

Student’s t-test was used to determine the significance of differences between two group means. Statistical significance among means of more than two groups was determined by one-way analysis of variance (ANOVA) followed by Dunnett’s test. Statistical significance was defined as p<0.05. There were no excluding data in any experiments.

## Results

### Effects of ingredients on the absorption of theanine

**Fig 1:**
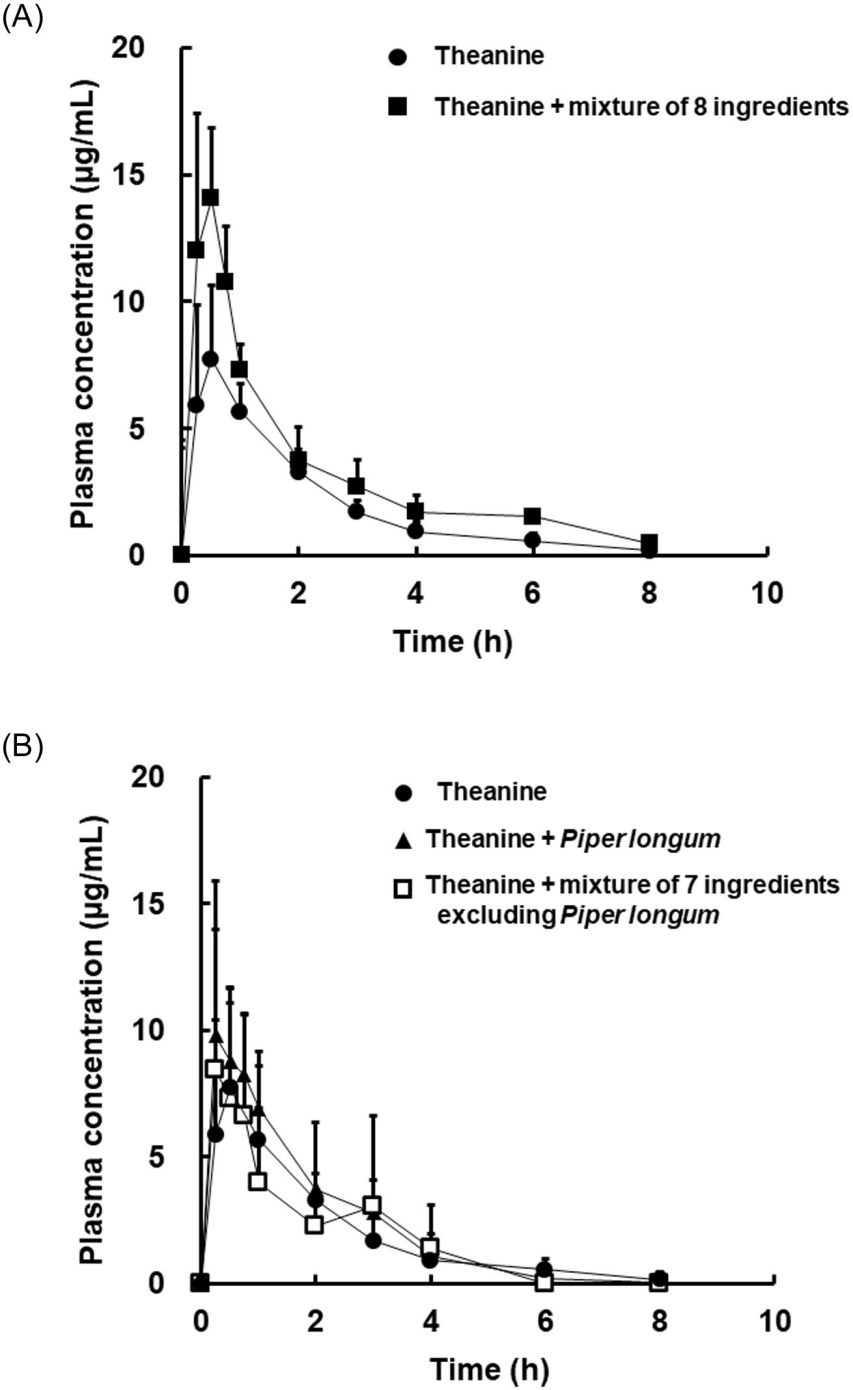
Plasma concentration of theanine after oral administration of theanine powder and/or a mixture of ingredients. Each point represents the mean with S.D. of 5–6 measurements. Powder of theanine in 0.5% methylcellurose (closed circle) and a mixture of theanine and eight ingredients (closed square) were administered (A). Powder of theanine in 0.5% methylcellurose (closed circle), a mixture of theanine and *Piper longum* (closed triangle), and a mixture of theanine and seven ingredients excluding *Piper longum* (open square) were administered (B). Blood samples were obtained up to 8 h after administration. The dose of theanine in all groups was 25 mg/kg body weight.

In the first part of this study, the plasma concentrations of theanine with and without a mixture of 8 ingredients were investigated up to 8 h after oral administration (Fig 1A). The concentration reached to a maximum concentration about 30 min after oral administration and theanine was eliminated slowly up to 8 h after administration. The concentration of theanine in the theanine + mixture of 8 ingredients group remained at a high level compared with that in the theanine powder group. There was no significant difference in the value of T_max_ (time at maximum concentration) between these groups (Table 1). The maximum concentration (C_max_) in the theanine + mixture of 8 ingredients group was 14.7 ± 4.2 μg/mL whereas the value in the theanine powder group was 9.3 ± 2.3 μg/mL, respectively. The values of AUC in the theanine powder group and theanine + the mixture of 8 ingredients group were 15.0 ± 4.1 and 26.0 ± 8.0, respectively. It was shown that the mixture of 8 ingredients significantly increased the absorption of theanine, extent of absorption, not rate of absorption.

**Table 1:** Pharmacokinetic parameters of theanine after oral administration of each formulation.

	C_max_ (μg/mL)	T_max_ (h)	AUC_0-8 h_ (μg×h/mL)	K_e_ (1/h)	T_1/2_ (h)
Theanine powder	9.3 ± 2.3	0.5 ± 0.3	15.0 ± 4.1	0.5 ± 0.1	1.4 ± 0.3
Theanine powder + 8 ingredients	14.7 ± 4.2*	0.5 ± 0.2	26.6 ± 8.0*	0.4 ± 0.1	1.7 ± 0.3
Theanine powder + *Piper longum*	11.3 ± 4.5	0.7 ± 0.6	19.7 ± 6.1	0.8 ± 0.3	1.0 ± 0.3
Theanine powder + 7 ingredients (excluding *Piper longum*)	11.9 ± 2.5	0.8 ± 1.0	14.9 ± 4.5	0.8 ± 0.3	1.0 ± 0.3

Each parameter represents the mean ± S.D. of 4–7 measurements. The value of AUC was calculated by the trapezoidal method from the data Fig 1A and 1B.

*; significantly different from theanine powder group at p<0.05 by one-way ANOVA followed by the Tukey-Kramer test.

It was then investigated the effects of the ingredients on the absorption of theanine. *Piper longum* in the ingredients was focused on since it contains piperine. It was hypothesized that piperine increases the absorption of theanine. Theanine + *Piper longum* was administered and the concentration was determined. At 15 min after administration, the plasma concentration of theanine was increased in the theanine + *Piper longum* group compared with that in the theanine powder group (Fig 1B). The value of AUC was not significantly different but tended to increase in the theanine + *Piper longum* group (Table 1). The effects of ingredients other than *Piper longum* were also investigated. There was little alteration in the plasma concentration profile in the theanine + mixture of 7 ingredients (excluding *Piper longum*) group compared with that in the theanine powder group. It was shown that these 7 ingredients did not contribute to the improvement in absorption of theanine.

It was speculated that theanine has high membrane permeability and that its absorption is dependent on intestinal blood flow. To confirm this speculation, cephalexin was also administered and the plasma concentration profiles were investigated as a preliminary test. The concentration of cephalexin reached to a maximum concentration about 1.5 h after oral administration and then cephalexin was eliminated gradually up to 8 h after administration (S1 Fig). The concentration of cephalexin in the cephalexin + 8 ingredients group remained at a high level compared with that in the cephalexin powder group. The values of C_max_ and AUC in the cephalexin + mixture of 8 ingredients group were larger than those in the cephalexin powder group, though the differences were not significant (S1 Table). The values of T_max_, T_1/2_ and K_e_ were almost the same in the two groups.

### Effects of ingredients on the uptake of theanine into Caco-2 cells

**Fig 2:**
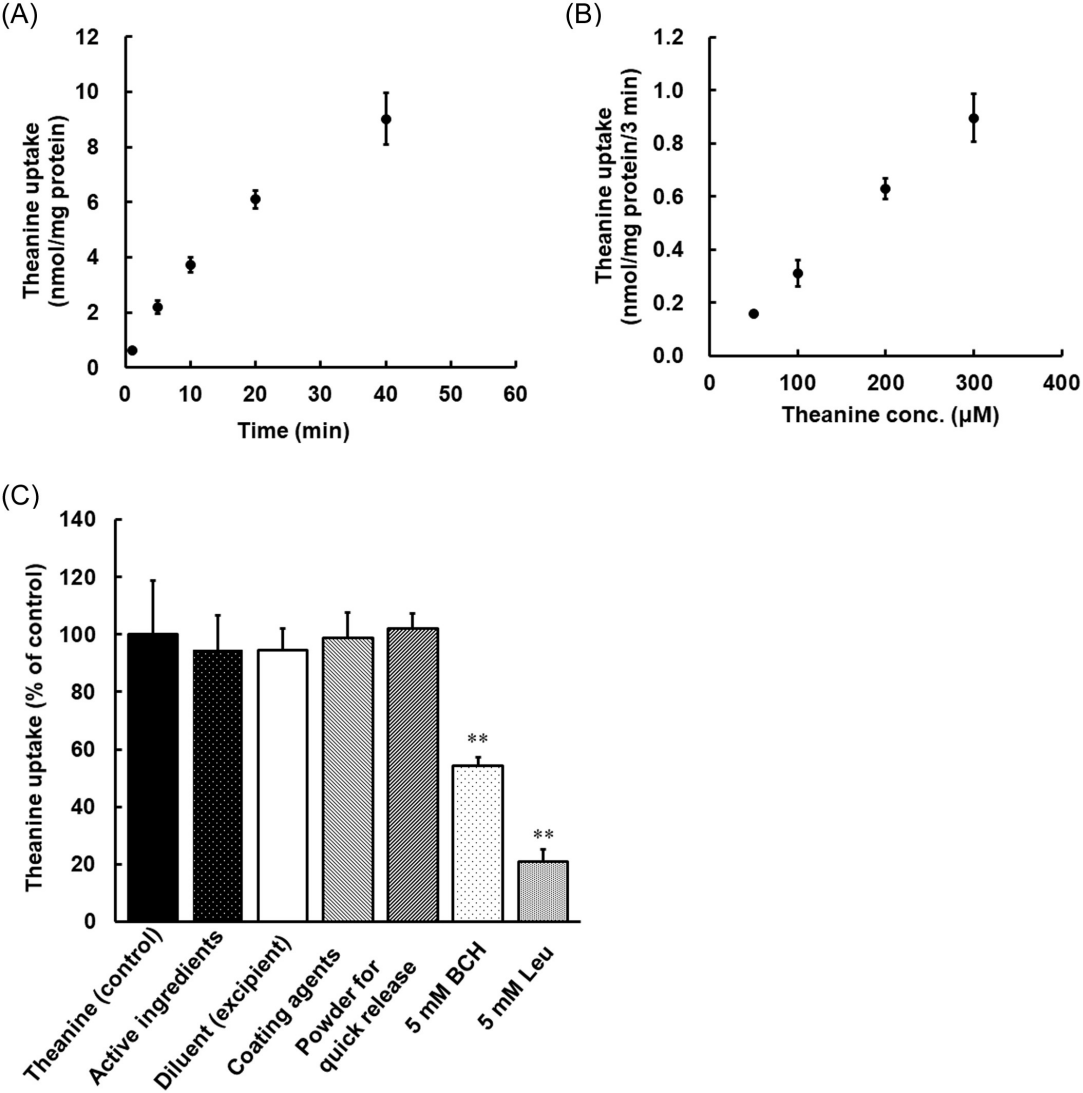
Time course (A) and concentration-dependence (B) of theanine accumulation and inhibitory effect of a mixture of ingredients and inhibitors of transporter on uptake of theanine (C) in Caco-2 cells. The uptake of theanine (500 μM) at 1, 5, 10, 20 and 40 min (A) and the accumulation of theanine (50, 100, 200 and 300 μM) (B) were measured at pH 7.4 at 37°C. The accumulation of theanine (200 μM) was measured at pH 7.4 at 37°C in the absence (control) and presence of a mixture of ingredients or inhibitors (5 mM of BCH or leucine) (C). The concentration of theanine was determined using an LC/MS. Each point represents the mean with S.D. of 3–4 measurements. **; significantly different from the control at p<0.01.

Theanine was shown to be mainly transported by the Na^+^-independent neutral amino acids system L transport pathway [12]. It was hypothesized that the ingredients strengthen the transporter activity. Caco-2 cells were used to investigate the effects of ingredients on the uptake of theanine. The time course of theanine accumulation is shown in Fig 2A. The accumulation of theanine increased for 40 min after the start of incubation. However, there was no linearity even at 5 min after the start of incubation. Thus, the initial accumulation was evaluated at 3 min. The concentration-dependence of theanine accumulation by Caco-2 cells was then investigated. It was confirmed that accumulation of theanine increased in a dose-dependent manner up to about 300 μM (Fig 2B). It was therefore evaluated the accumulation of theanine at 200 μM since that concentration was sufficient to determine the accumulation. It was then investigated whether the transporter influenced on the uptake of theanine into Caco-2 cells using BCH and leucine, inhibitors of the system L transport system. BCH and leucine significantly inhibited the uptake of theanine into Caco-2 cells by 50% and 80%, respectively (Fig 2C). On the other hand, there was no significant difference in the accumulation of theanine between the control group and mixture of 8 ingredients group.

### Evaluation of intestinal blood flow by fluorescence imaging using ICG

**Fig 3:**
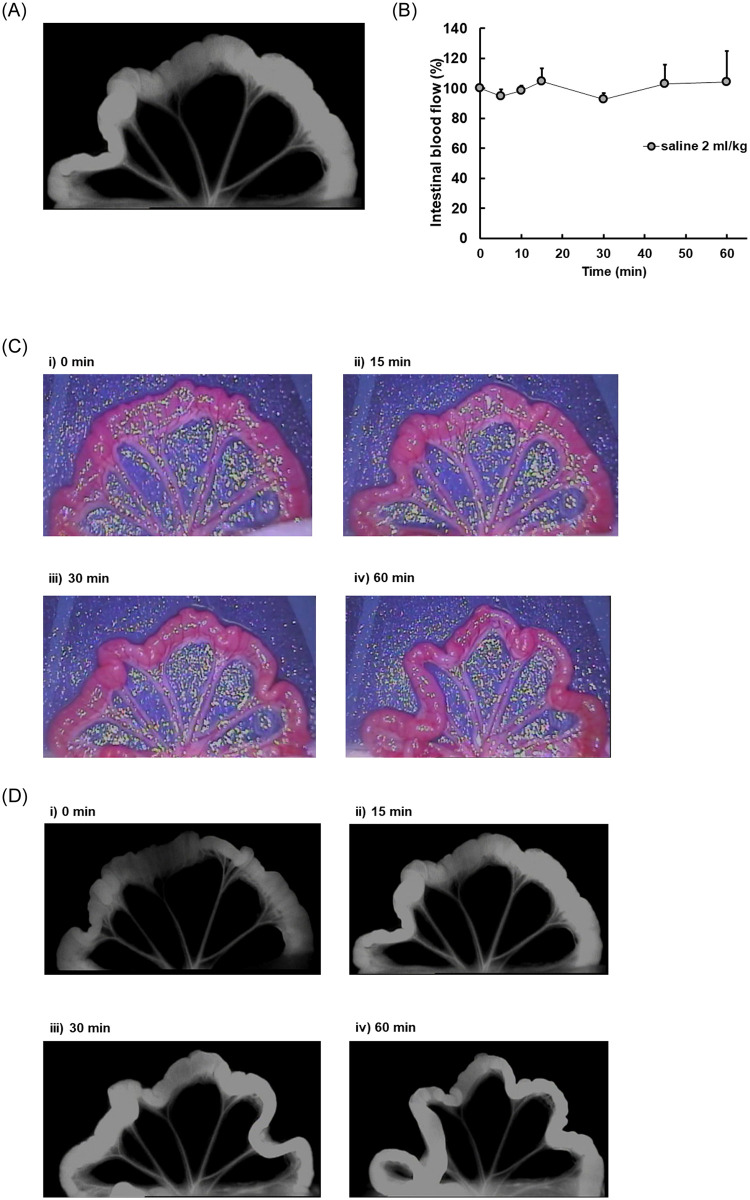
Intestine and mesentery after injection of ICG (A) and time course of intestinal blood flow after injection of saline (B). Visualization of the intestine after administration of theanine and a mixture of ingredients in a bright field (C) and a dark field (D) at 0, 15, 30 and 60 min. The three-branched part of blood vessels present in the mesentery (arrows) was selected for evaluation of the fluorescence intensity in each rat (A). ICG was administered by constant intravenous infusion from the tail vein at 5 mL/h with a syringe driver. At 15 min after the start of injection, fluorescence time lapse imaging was obtained for 30 sec with pde-neo C10935-20. Saline was then administered into the intestine and fluorescence time lapse imaging was also obtained for 1 h after administration. The average of the fluorescence intensity for 30 sec from the start of imaging was calculated with ImageJ^®^. Each point represents the mean with S.D. of 3 measurements (B). Appearances of the intestine in bright (C) and dark (D) fields at (i) 0, (ii) 15, (iii) 30 and (iv) 60 min after administration of a mixture of ingredients were obtained. Each appearance was shown as a representative of the three measurements.

It was focused on the use of ICG as a means for visually evaluating the possible alteration of intestinal blood flow caused by these ingredients. ICG was injected continuously from the tail vein and a steady state was confirmed (Fig 3A and S2 Fig). The physiological condition of the intestine must be maintained, although the intestine was exposed to the outside of abdominal cavity in this study. Saline was then administered into the intestine as a control. There was no alteration in fluorescence intensity of the intestine up to 60 min after administration (Fig 3B). It was also confirmed that the physiological condition and the peristalsis of the intestine were maintained throughout the experiments (Fig 3C and 3D).

### Effects of piperine and some ingredients on intestinal blood flow

**Fig 4:**
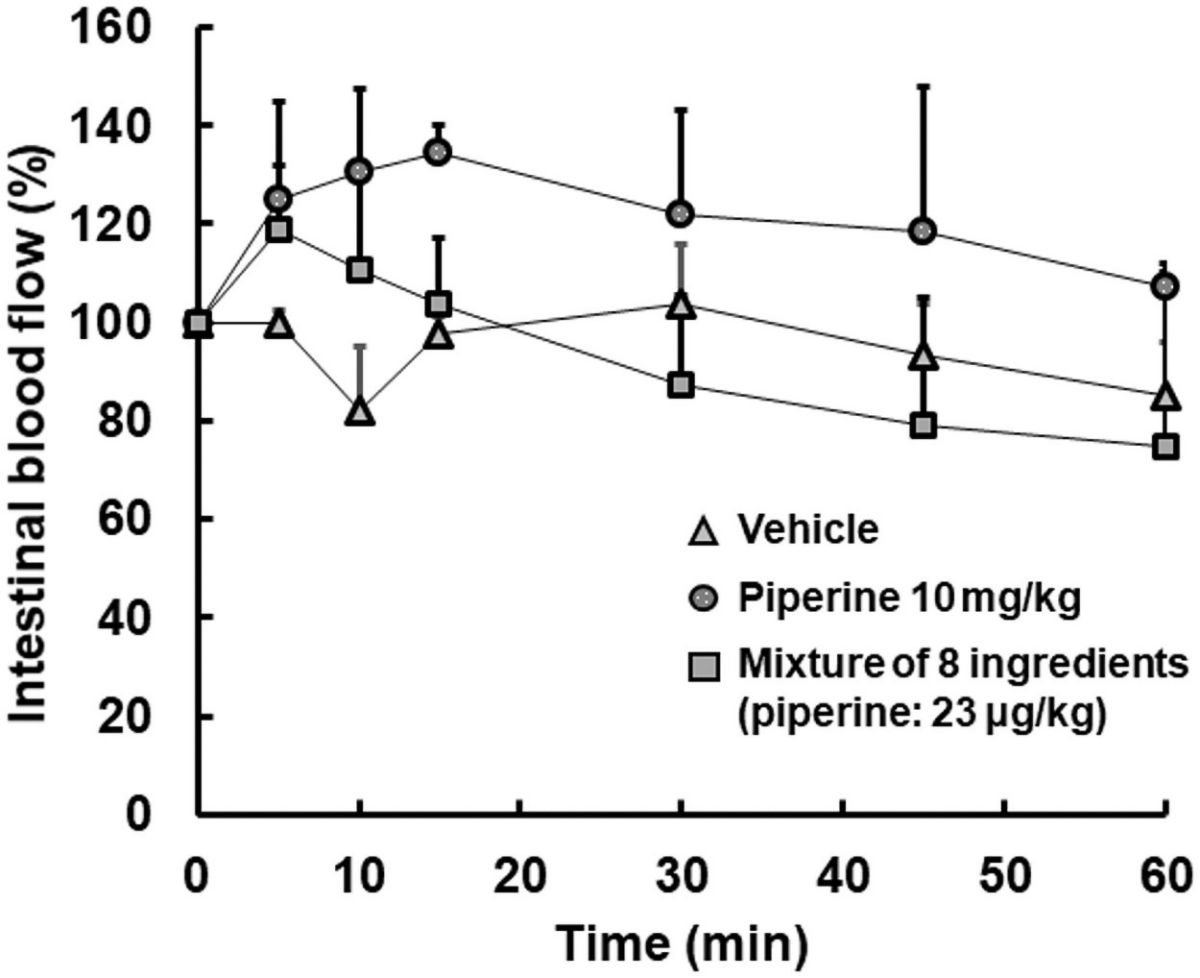
Time course of intestinal blood flow after administration of a vehicle or piperine and a mixture of ingredients. ICG was administered by constant intravenous infusion from the tail vein at 5 mL/h with a syringe driver. At 15 min after the start of injection, fluorescence time lapse imaging was obtained for 30 sec with pde-neo C10935-20. A vehicle (ethanol/propylene glycol/distilled water = 1/7/30 (v/v/v)) (triangle) or piperine (10 mg/kg) (circle) or a mixture of ingredients (dose of piperine: 23 μg/kg) (square) was then administered into the intestine and fluorescence time lapse imaging was also obtained for 1 h after administration. The average of fluorescence intensity for 30 sec from the start of imaging was calculated with ImageJ^®^. Each point represents the mean with S.D. of 3 measurements.

It was next investigated the effects of piperine and some ingredients on intestinal blood flow (Fig 4). There was little alteration in the ratio of intestinal blood flow up to 60 min after injection in the vehicle group and the saline group. On the other hand, the intestinal blood flow was confirmed to increase largely up to 15 min after injection in the piperine (10 mg/kg: positive control). The mixture of 8 ingredients including piperine (23 μg/kg) also increased intestinal blood flow in the first of 15 min after injection and the increased intestinal blood flow decreased slowly. It was shown that the mixture of 8 ingredients increased the absorption of theanine via an increase in intestinal blood flow.

## Discussion

L-theanine is a component of green tea that has various functions. Various supplements formulations of theanine have been developed. It is known that additives in supplements and foods often affect the absorption of the main component, but the detailed mechanisms by which absorption of main component is affected have not been elucidated in many cases. We previously showed some specific additives, oleyl polyethylene acetic acids, increased the absorption of coenzyme Q10 [25]. In this study, the effect of ingredients in the formulation on the absorption of theanine was investigated. A new imaging method for detecting change in blood flow was also developed to clarify the effect in detail.

In the first part of this study, the plasma concentration profile of theanine after oral administration of theanine powder was confirmed. The concentration reached to a maximum concentration about 30 min after oral administration and then theanine was eliminated gradually up to 8 h after administration. These results are consistent with the results of a previous report [10]. It was then confirmed the effects of ingredients other than theanine on the plasma concentration of theanine. Eight ingredients significantly increased the plasma concentration of theanine compared with that in the case of administration of theanine powder, not rate of absorption of theanine (Fig 1A, Table 1). In these ingredients, we focused on piperine, which is known as an enhancer of the absorption of various components, in *Piper longum* [26,27]. Piperine is known to be a potent inhibitor of cytochrome P450, particularly CYP3A4 and CYP1A2 [28]. Piperine is also known as an inhibitor of glucuronidase [28,29]. It was reported that theanine was hydrolyzed into glutamic acid and ethylamine [10]. It was considered that CYP and glucuronidase are not probably involved in theanine metabolism. Therefore, an increased in blood flow is considered as another mechanism of increased theanine absorption.

It was next hypothesized that piperine affected the blood flow and increased the absorption of theanine. The plasma concentration of theanine increased in the theanine + *Piper longum* group compared with that in the theanine powder group (Fig 1B). The value of AUC was not significantly different but tended to increase in the theanine + *Piper longum* group (Table 1). On the other hand, there was little alteration of the plasma concentration profile in the theanine + mixture of 7 ingredients (excluding *Piper longum*) group compared with that in the theanine powder group. It was shown that the mixture of 7 ingredients did not contribute to the increase of absorption of theanine but that the mixture of 8 ingredients including *Piper longum* increased theanine absorption in a synergistic manner.

Theanine has been shown to be transported rapidly by the Na^+^-independent neutral amino acids system L transport pathway [12]. It was speculated that theanine has high membrane permeability and that its absorption is dependent on intestinal blood flow. It was also hypothesized that the mechanism by which the absorption of theanine is improved is an increase in intestinal blood flow. To confirm the hypothesis, cephalexin was then administered to rats. It was not needed to consider the influence of inhibition of metabolism because cephalexin is excreted into urine as an unchanged form. Cephalexin has high membrane permeability and is transported into blood by PEPT1 (peptide transporter 1) [30]. The plasma concentration profile of cephalexin after oral administration was obtained at the start time of 14:00 in all experiments since there is a circadian variation in the expression of PEPT1 [31]. The concentration of cephalexin in the cephalexin + 8 ingredients group remained at a high level compared with that in the cephalexin powder group (S1 Fig). The values of C_max_ and AUC in the cephalexin + mixture of 8 ingredients group were larger than those in the cephalexin powder group, though the differences were not significant (S1 Table). These results indicated that the mixture of 8 ingredients including *Piper longum* increased the absorption of cephalexin as well as that of theanine. The results suggested that these ingredients increased intestinal blood flow.

It was next hypothesized that the ingredients strengthen the transporter activities. The effects of the ingredients on the uptake of theanine were investigated using Caco-2 cells. BCH and leucine inhibited the uptake of theanine into Caco-2 cells by 50% and 80%, respectively (Fig 2C). On the other hand, there was no significant difference in the accumulation of theanine between the control group and the mixture of 8 ingredients group. These results suggested that the mixture of 8 ingredients did not contribute to the activity of the transporter. The results also suggested that the limiting factor of the increase in absorption of theanine is based on an increase in intestinal blood flow rather than permeation of the cell membrane.

*Piper longum* and piperine have been reported to increase the bioavailability of some components [29]. Their group reported that the mechanism would promote rapid absorption from the gastrointestinal tract and protect the components from being metabolized in its first passage though the liver after being absorbed. Another group also reported the mechanism of Trikatu and individual components and piperine [32]. However, those results do not directly demonstrate that *Piper longum* or piperine activate the intestinal movements and effects on the blood flow. Kono et al. reported that colonic blood flow was measured by laser Doppler tissue blood flowmetry [33]. The colonic vascular conductance was calculated as the quotient of mean blood flow divided by mean arterial blood pressure. It was then focused on the use of ICG and set out to establish the evaluation of the alteration of possible intestinal blood flow by these ingredients visually and quantitatively. It is possible to observe blood flow distribution, leakage to tissues lymphatic flow in the body and from the body surface in take advantage of property that ICG emits fluorescence when irradiated specific excitation light [34]. Injected ICG is distributed only in blood and is excreted into bile [34]. It was speculated that we could evaluate intestinal blood flow as fluorescence intensity since a region with large blood flow increases fluorescence intensity based on the properties of ICG. There have been few studies in which intestinal blood flow was evaluated directly with ICG. Behrendt et al. observed the intestinal transit using ICG though no time-profiles after intestinal treatment was shown [35]. Wada et al. also reported that colonic blood flow was analyzed using ICG and some parameters were calculated [36]. There were some differences of parameters in their report. However, it was considered necessary to observe several time points after oral administration to see the effects of orally administered ingredients on the intestine. A steady state of ICG and no alteration in fluorescence intensity of the intestine up to 60 min after administration of saline were confirmed (Fig 3B and S2 Fig). The physiological condition and peristalsis of the intestine through experiments had also checked (Fig 3C and 3D). Then the effects of piperine and some ingredients on intestinal blood flow were investigated (Fig 4). A positive control of piperine (10 mg/kg) based on previous reports [14,25] increased intestinal blood flow up to about 135% for 15 min after the start of injection. Eight ingredients including piperine (23 μg/kg) also increased intestinal blood flow to about 120% in the first of 15 min after the start of injection and the increased intestinal blood flow then decreased gradually up to 60 min. The extent of enhancement of intestinal blood flow by the eight ingredients including piperine was smaller than that by the positive control, though consideration should be given to the small doses (about 1/500).

As a limitation of this study, it is difficult to explain to rate of increased absorption of theanine only by increasing blood flow. In addition to this increased blood flow, other mechanisms may be involved in the increased absorption of theanine by these 8 ingredients. Further studies are needed to clarify these mechanisms and contribute to evidence-based formulation development.

## Conclusion

Plasma concentration of theanine increased significantly in the group with theanine and a mixture of 8 ingredients including *Piper longum*. The effect of piperine in the *Piper longum* on the blood flow was focused to investigate the mechanism in detail. It succeeded in visual and quantitative evaluation of intestinal blood flow using ICG in physiological conditions. It was also confirmed improvement in the absorption of theanine and enhancement of intestinal blood flow by the eight ingredients including piperine. These results could be applied to elucidate the absorption mechanism of other components based on the scientific evidence. Further studies to apply this system to evaluation of the intestinal blood flow are in progress.

## Supporting information

S1 Fig Plasma concentration of cephalexin after oral administration of theanine powder and/or a mixture of ingredients. The dose of cephalexin in both groups was 10 mg/kg body weight. Each point represents the mean with S.D. of 5 measurements. Powder of cephalexin in 0.5% methylcellurose (closed circle) and a mixture of theanine and eight ingredients (open circle) were administered.
(TIF)

S2 Fig Confirmation of a steady state of ICG for 30 sec at 15 min after injection. ICG was administered by constant intravenous infusion from the tail vein at 5 mL/h with a syringe driver. At 15 min after the start of injection, fluorescence time lapse imaging was obtained for 30 sec with pde-neo C10935-20. The fluorescence intensity from time lapse imaging was quantified with ImageJ^®^. This is the state before the administration of test solution.
(TIF)

S1 Table Pharmacokinetic parameters of cephalexin after oral administration of each formulation. Each parameter represents the mean ± S.D. of 5 measurements. The value of AUC was calculated by the trapezoidal method from the data S1 Fig.
(DOCX)
