# Supplementary figures and images for: An imaging approach for determining the mechanism of enhancement of intestinal absorption of an L-theanine supplement
Source: PLoS One. 2021 Jun 11;16(6):e0253066. doi: 10.1371/journal.pone.0253066 (PMC8195392; doi:10.1371/journal.pone.0253066)

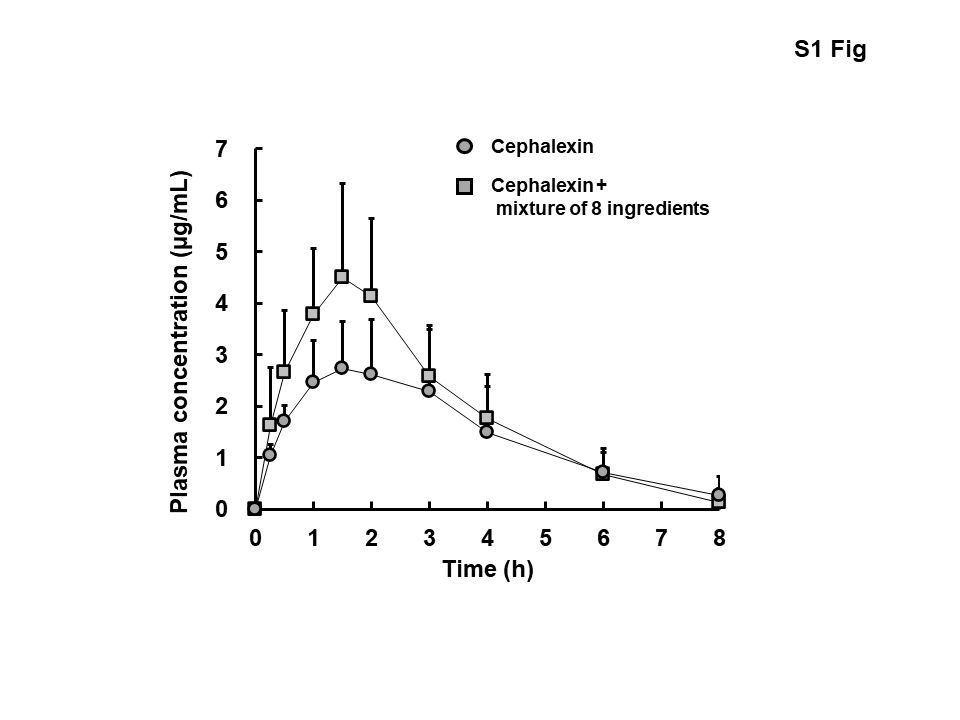

Supplement: S1 Fig — The dose of cephalexin in both groups was 10 mg/kg body weight. Each point represents the mean with S.D. of 5 measurements. Powder of cephalexin in 0.5% methylcellurose (closed circle) and a mixture of theanine and eight ingredients (open circle) were administered. (TIF) [file pone.0253066.s001.tif]

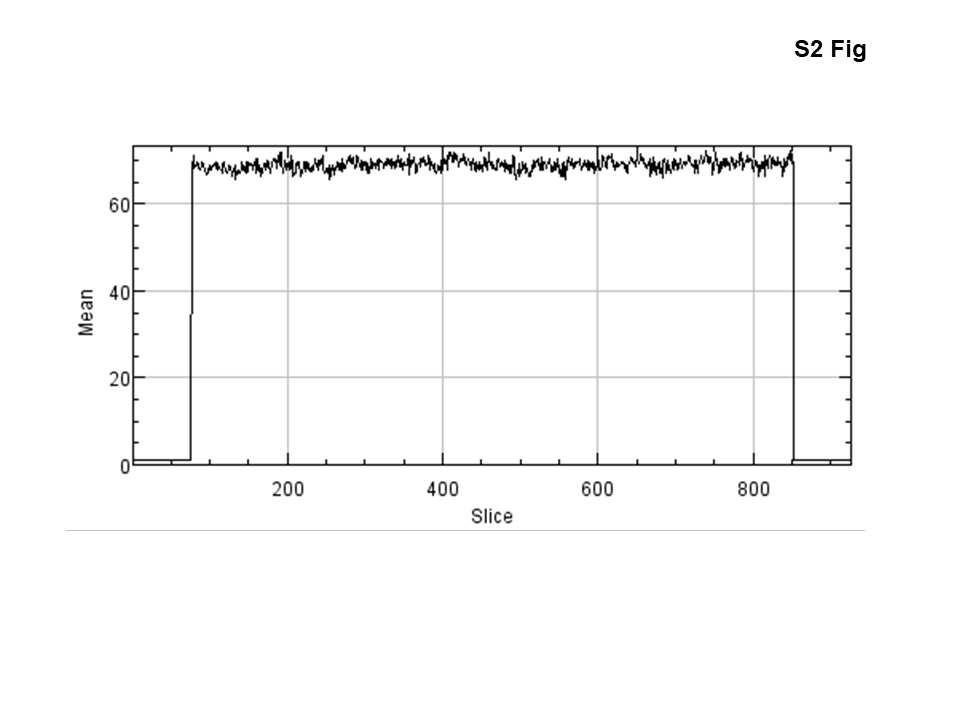

Supplement: S2 Fig — ICG was administered by constant intravenous infusion from the tail vein at 5 mL/h with a syringe driver. At 15 min after the start of injection, fluorescence time lapse imaging was obtained for 30 sec with pde-neo C10935-20. The fluorescence intensity from time lapse imaging was quantified with ImageJ®. This is the state before the administration of test solution. (TIF) [file pone.0253066.s002.tif]
