# Supplementary material for: An imaging approach for determining the mechanism of enhancement of intestinal absorption of an L-theanine supplement
Source: PLoS One. 2021 Jun 11;16(6):e0253066. doi: 10.1371/journal.pone.0253066 (PMC8195392; doi:10.1371/journal.pone.0253066)
Supplement: S1 Table — Each parameter represents the mean ± S.D. of 5 measurements. The value of AUC was calculated by the trapezoidal method from the data S1 Fig. (DOCX) [file pone.0253066.s003.docx]

|  | C_max_  (μg/mL) | T_max_ (h) | AUC_0-8 h_  (μg×h/mL) | K_e_ (1/h) | T_1/2_ (h) |
| --- | --- | --- | --- | --- | --- |
| Cephalexin powder | 3.1 ± 1.0 | 2.0 ± 0.9 | 11.7 ± 5.2 | 0.4 ± 0.2 | 2.4 ± 1.8 |
| Cephalexin powder + 8 ingredients | 5.0 ± 1.6 | 1.5 ± 0.6 | 15.4 ± 4.4 | 0.4 ± 0.2 | 1.8 ± 0.9 |
